# Journey to an Improved Quality of Life: Filarial Lymphoedema and Physiotherapy Rehabilitation

**DOI:** 10.7759/cureus.48403

**Published:** 2023-11-06

**Authors:** Mansee Dangare, Tejaswini Fating

**Affiliations:** 1 Department of Community Health Physiotherapy, Ravi Nair Physiotherapy College, Datta Meghe Institute of Higher Education and Research, Wardha, IND

**Keywords:** lymphatic drainage, physiotherapy rehabilitation, vascularized lymph node transfer, lymphoedema, elephantiasis

## Abstract

Filarial lymphoedema is a tropical parasitic worm illness caused mostly by *Wuchereria bancrofti*. This disease impacts millions of individuals in endemic areas, causing significant impairment and financial discomfort. Elephantiasis is a chronic lymphatic drainage failure causing swelling in the legs and genitalia. It is a chronic condition characterized by the buildup of lymphatic water, causing pain and reduced limb activity. Genetic abnormalities, trauma, surgery, infection, or cancer cause the disease. Treatment options include antiparasitic drugs, surgical interventions, and nonsurgical interventions like compression therapy. Lymphatic system transplantation, liposuction, and vascularized lymph node transfer are surgical procedures that restore lymphatic circulation and reduce swelling, potentially lifesaving for individuals with lymphedema who have not responded well to conservative treatment. The present case report concerns a 30-year-old male with a history of chronic lower leg elephantiasis for 17 years. Duplex color Doppler revealed a large, well-defined collection in the right foot with a heterogeneous group with few solid cystic components suggestive of a solid cystic mass lesion. MRI showed diffuse subcutaneous edema in the dorsum, medial, and lateral aspect of the foot with the medial, lateral, and posterior aspect of the leg appearing hyperintense on T2/proton density spectral attenuated inversion recovery (PDSPAIR), and hypointense on T1. Dilated lymphatic channels were seen coursing through in the cutaneous plane. The patient underwent a comprehensive surgical procedure that included excision of fibrotic tissue and subcutaneous adipose tissue, followed by lymph node transfer. Following the surgeon's clearance, a targeted early physiotherapy intervention could normalize functional potencies and help in recovery. Post-treatment changes such as reduction in lymphoedema, strength, and mobility, which are essential for patients with the activity of daily living, were observed.

## Introduction

Lymphatic filariasis (LF), a condition where adult worms are detected in the lymphatic system, is the most common kind of filariasis and is also referred to as elephantiasis [1]. Although the worldwide prevalence of filariasis is 20% (about 119 million cases), the disease remains of significant local relevance in India. The global incidence of LF is around 1.3%. Morbidity in LF manifests itself as painful and disfiguring obvious chronic symptoms such as lymphoedema (acute dermato-lymphangio-adenitis (ADLA) and elephantiasis) and male urogenital illness (hydrocele and lymphatic scrotum) [2]. Filarial worms or filariae belong to the Filarioidea superfamily of highly specialised parasitic nematodes that live in human tissue and blood vessels. The condition causes a variety of indicators, largely because mature worms inhabit and clog lymphatic arteries [1].

Lymphedema is a high-protein edema that develops when the lymphatic system becomes persistently overburdened. Initially, fluid-rich phases evolve slowly towards subcutaneous section expansion and fibrosis, as well as skin excessive keratin (elephantiasis) [3]. Lymphoedema significantly impacts the quality of life of LF patients, leading to physical, functional, psychological, financial, and social issues [4]. Lymphoedema exercises and limb elevation help relieve swollen limbs and prevent further swelling. These exercises teach leg muscles to pump fluid, prevent joint stiffening, and minimize lymphatic fluid collection. Leg raising also helps reduce fluid migration along the vertical plane. Treatment of lymphoedema resulting from LF, which includes dermatological treatments and basic activities, is a successful approach for improving patients' standard of living [5]. Lymphedema caused by LF is a debilitating disorder that usually appears during puberty [6]. Numerous community-based epidemiologic studies and individual case reports confirm the presence of LF infection in children and the presence of clinically apparent illness [7]. Adult filarial worms live in the lymphatics and lymph nodes, where they cause distortion and hypertrophy of the lymphatic channel membranes [8].

Treating LF-related psychological difficulties should include basic and successful hygienic interventions such as periodic foot cleaning using soap and water and daily leg movements [9]. Filariasis prophylaxis is critical in avoiding disability. Therapy for filariasis patients encompasses physical, mental, community, and financial components. Health issues and rehabilitation maintenance require successful rehabilitation [5]. Tight bandaging effectively eradicates lymphoedema in filarial elephantiasis of the leg, resulting in immediate relief and the cessation of repeated lymphangitis episodes [10]. 

## Case presentation

Patient information

A 30-year-old male patient presented in July 2023 with a chief complaint of edema in the right foot. He first started experiencing itching and swelling, which was sudden in the beginning, over both lower limbs when he was four years old, which was worsened in seasonal variation and relieved after some days. These episodes continued until he was 13 years old, and after that, as the swelling around the right lower foot was not subsiding, he visited a local hospital in Gadchiroli, Maharashtra, India, where investigations were done, and he was given medications but remained undiagnosed; this continued after some years and in 2020, he noticed an increase in the growth of mass over the right foot. In March 2023, he went to a government hospital with the same chief complaint, where an investigation was done, and an operation was suggested. Due to his poor financial condition, the patient took only medication and applied a crepe bandage. The situation worsened and this led to his current presentation. 

Investigations were done including duplex color Doppler and MRI of the right leg. Doppler revealed a large, well-defined collection in the right foot with a heterogeneous collection with few solid cystic components suggestive of a solid cystic mass lesion. MRI revealed diffuse subcutaneous edema in the dorsum, medial, and lateral aspect of the foot, medial and lateral and posterior aspect of the leg, appearing hyperintense on T2/Proton density spectral attenuated inversion recovery (PDSPAIR), hypointense on T1. Dilated lymphatic channels were seen coursing through in the cutaneous plane. of the right leg, which revealed a large, well-defined collection in the right foot with a heterogeneous group of solid cystic components, suggestive of a solid cystic mass lesion. It was diagnosed as lymphoedema caused by elephantiasis.

**Figure 1:**
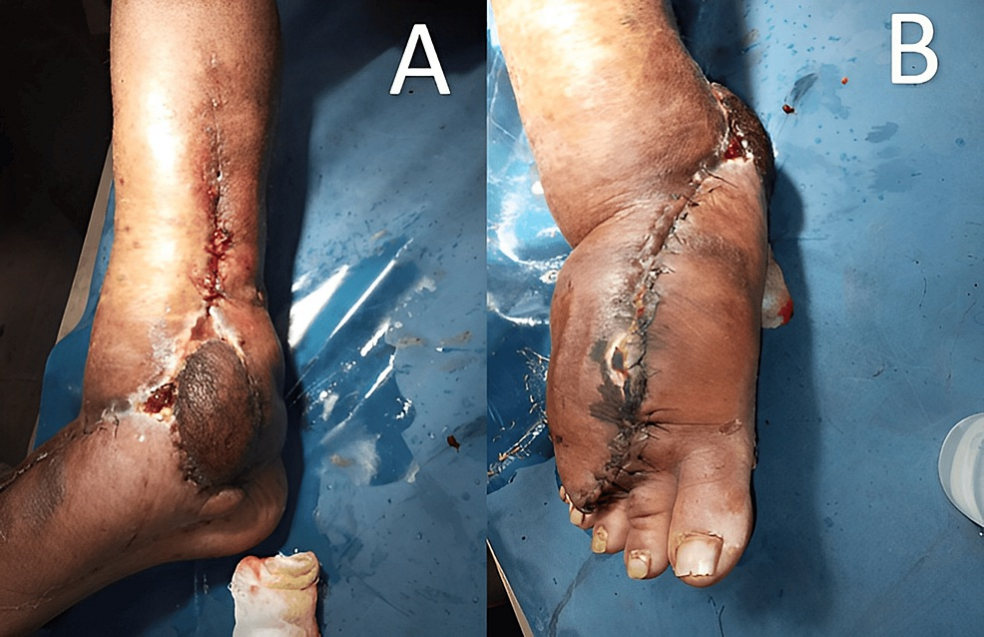
Postoperative images showing: (A) Medial aspect of foot and leg; (B) Anterior aspect of foot

Vascularized right submental lymph node transfer was done for right lower limb filarial lymphedema. Postoperative images are shown in Figure 1. 

Postoperative clinical findings

Postoperatively, the patient was conscious and well-oriented. On inspection, it was observed that both the patient’s legs were extended and slightly abducted, and the right leg was supported with a pillow and elevated to 15 degrees. The bandage was seen on the right leg from mid-lower leg to foot with visible swelling proximally to the knee joint. On palpation, local skin temperature was raised, grade 2 edema was present, and pain was present at the operative site. The range of motion of all joints is shown in Table 1, and the girth measurement is shown in Table 2. The visual analog scale (VAS) scored 7/10 on activity and 2/10 on rest.

**Table 1:** Range of motion Rt: Right side; Lt: Left side

Joint	Rt Active	Rt Passive	Lt Active	Lt Passive
Hip				
Flexion	0-110^o^	0-115^o^	0-120^o^	0-120^o^
Extension	0-20^o^	0-25^o^	0-30^o^	0-30^o^
Abduction	0-40^o^	0-45^o^	0-45^o^	0-45^o^
Adduction	0-20^o^	0-25^o^	0-30^o^	0-30^o^
Knee				
Flexion	0-125^o^	0-130^o^	0-135^o^	0-135^o^
Ankle				
Plantar flexion	0-25^o^	0-30^o^	0-50^o^	0-50^o^
Dorsiflexion	0-10^o^	0-15^o^	0-20^o^	0-20^o^

**Table 2:** Girth measurement

Girth Measurement	Affected Side (Right)	Non-affected Side (Left)	Difference
From the base of the patella (quadriceps) 2 inch, 4 inch, 6 inch, respectively	32 inch, 32 inch, 35 inch, respectively	33 inch, 33 inch, 36 inch, respectively	1 inch, 1 inch, 1 inch, respectively
From apex of patella (calf muscles)	36 inch, 39 inch, 41 inch, respectively	30 inch, 31 inch, 32 inch, respectively	6 inch, 8 inch, 9 inch, respectively

Sensory examination

Postoperative superficial, deep, and cortical sensations are given in Table 3.

**Table 3:** Sensory examination

Sensation	Upper Limbs		Lower Limbs	
Superficial	Right (Affected side)	Left (nonaffected side)	Right (affected side)	Left ( nonaffected side)
Pain	intact	intact	intact	intact
Touch	intact	intact	intact	intact
Temperature	intact	intact	intact	intact
Deep				
Vibration	intact	intact	intact	intact
Kinaesthesia	intact	intact	intact	intact
Proprioception	intact	intact	intact	intact
Cortical				
2-point discrimination	intact	intact	intact	intact
Stereognosis	intact	intact	intact	intact
Barognosis	intact	intact	intact	intact

Medical management

Medicine and dosage, along with its duration, are given in Table 4.

**Table 4:** Dosage and duration of prescribed medication Tab: tablet; Syp: syrup; mg: milligrams; BD: twice a day; OD: once a day; TDS: thrice a day.

Medication	Dosage	Duration
Tab. Augmentine	625 mg	TDS
Tab. Pantop	40 mg	OD
Tab. Zerodol SP	650 mg	OD
Syp. Duphalac	30 ml	OD
Inj. Clexane	0.4 mg	OD
Tab. Doxycycline	100 mg	BD

Physiotherapy

Postoperatively, the patient underwent physiotherapy rehabilitation. The timeline of events till the start of physiotherapy is given in Table 5.

**Table 5:** Timeline of events

Event	Time
First time itching over both legs	When he was 4 years old
Recurrence of symptoms	When he was 13 years old
Presented to the government hospital	April 17, 2023
Presented to the current hospital	June 10, 2023
Date of admission in current hospital	July 31, 2023
Date of surgery	August 12, 2023
Starting date of physiotherapy	August 13, 2023

**Figure 2:**
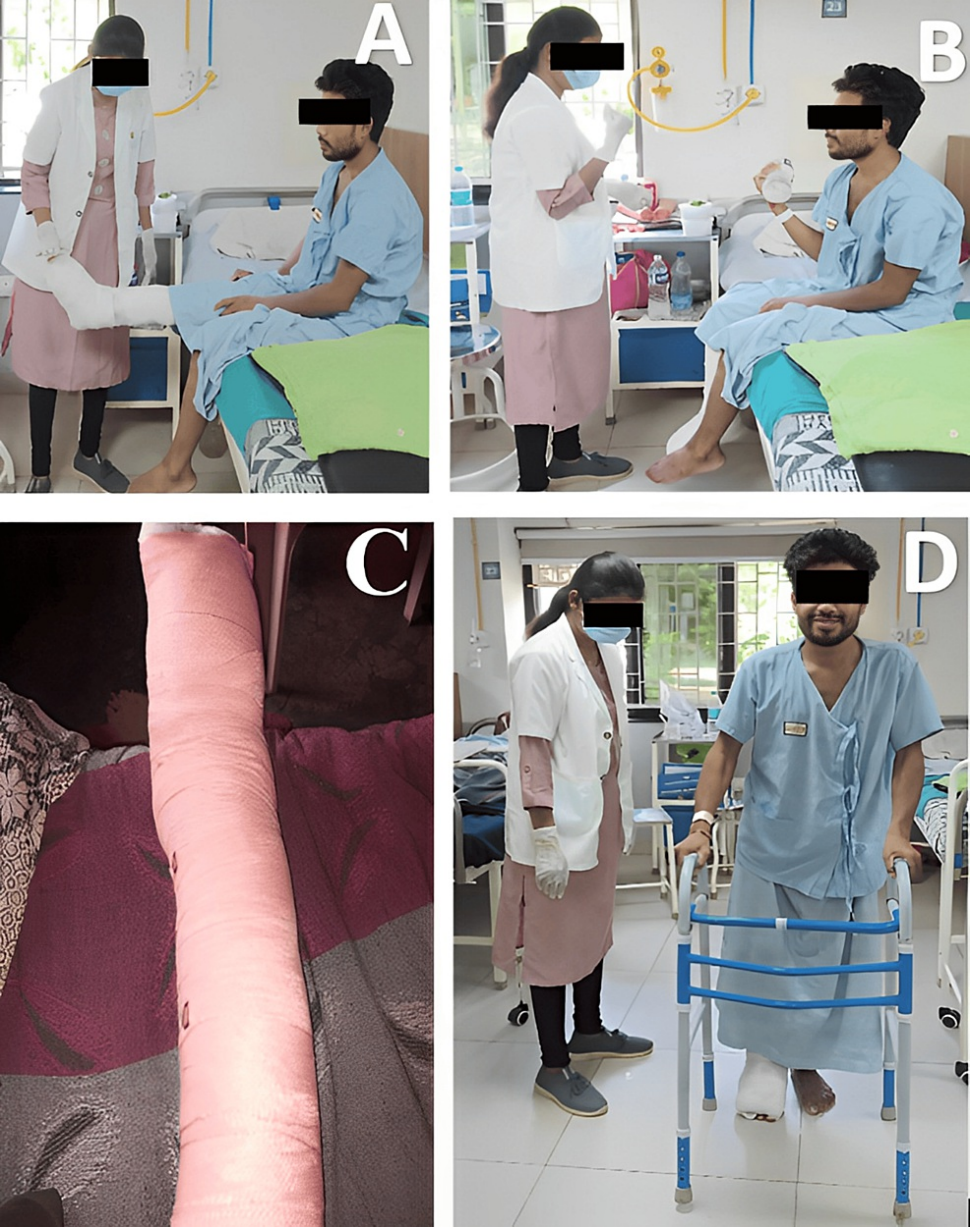
Physiotherapy including (A) dynamic quads, (B) upper limb strengthening exercise with 1 liter water bottle, (C) multilayered crepe bandage on the right foot, and (D) gait training session with the help of walker

The goal-oriented physiotherapy protocol is given in Table 6. Physiotherapy sessions are shown in Figure 2. Physiotherapy lasted for four to six weeks followed by home exercise program after discharge.

**Table 6:** Goal-oriented physiotherapy protocol

Sr. No	Physiotherapy goals	Therapeutic Intervention	Treatment regimen
1.	To inform the patient and his relatives regarding the present surgical condition	Patients and relatives must be aware of the need for well-planned treatment, skincare, as well as hygiene.	Teaching for earlier mobility, placement of lower limbs, and resumption of tasks associated with everyday routine.
2.	To reduce limb oedema	Multilayered crepe bandage with the surged faradic current was given on the right foot with the figure-of-8 bandaging procedure (Figure 2C).	Every 2-3 hours each day.
3.	To recover ankle range of movement	Initially, active toe movements were initiated in dressing from the first day of surgery; after the removal of the bandage, ankle pumping exercises were initiated.	A single set of 10 repetitions two times each day.
4.	To reduce lymphoedema	Limb elevation of the right foot with support of pillow.	Every 2-3 hours each day
5.	Active strengthening of upper limb (Figure 2B)	Active assisted range of motion exercises of the upper limb with a one-liter water bottle for upper limb strengthening to regain the muscle tone, essential for ambulation with a walker. Then progress to 1 kg weight cuff.	A single set of 10 repetitions two times each day.
3.	To improve bed mobility exercises	1. Pelvic bridging, 2. Heel slides, 3. Rolling, 4. Dynamic Quads (Figure 2A), and 5. Bedside sitting.	A single set of 10 repetitions two times each day.
6.	To prevent respiratory and chest complications	Techniques like deep breathing, pursed lip breathing exercises, thoracic mobility exercises, and thoracic expansion exercises.	A single set of 10 repetitions two times each day.
7.	Gait training	The patient uses an assistive device like a walker for ambulation with partial weight bearing on the right foot (Figure 2D).	Walk within a hall (one round)

Table 7 shows pre- and post-physiotherapy outcome measures.

**Table 7:** Pre- and post-physiotherapy outcome measures

Outcome Measures	Pre-intervention	Post-intervention
Lymphatic Filariasis Specific Quality of Life Questionnaire	31/100	80/100
Visual Analog Scale	7/10	2/10

## Discussion

The initial treatment for long-term lymphoedema comprises a multimodal approach involving skin care, weight loss, pressure treatment, and rehabilitation. These treatments are useful at every phase of lymphoedema [11]. Mousavi and Mehdikhah proposed that operative surgery be explored following a six-month test of conservative therapy [12]. The basic idea behind surgical treatments is to remove extra tissue to reduce the size of the extremities. Surgical techniques that included subcutaneous fat and fibrous tissue excision and lymphatic surgery had minor results that were not sustained throughout the series [13].

LF is considered to be one of the "neglected diseases." Nevertheless, its abolition is achievable provided treatment is continued over five years, and the program offers unique traits that allow it to appeal to a diverse donor base. LF eradication may be helpful to the attainment of the Millennium Development Goals and serves as an unexpected worldwide health success story; initial objectives for increasing therapy numbers were successfully met [14]. Chronic filarial illness has severe social and financial consequences. Elephantiasis and hydrocele patients are frequently socially and economically marginalized. Immediate and long-term illnesses reduce economic production and exacerbate poverty [15].

Filariasis may appear as asymptomatic/subclinical microfilaremia, sudden illness with lymphadenitis or lymphangitis, or a long-term condition with lymphedema or elephantiasis. The gender of a person has a significant impact on the occurrence of long-term disease in LF; men are more likely than females to develop long-term pathologies [16]. The fundamental mechanism stays the same: the constant occlusion of lymphatic pathways, leading to lymphatic stasis, fibroblast development stimulation, lymph node destruction, lymphedema, and elephantiasis [17].

## Conclusions

The case of chronic lymphedema secondary to elephantiasis from a physiotherapy perspective underscores the critical role of physiotherapists in the holistic management of this complex and challenging condition. By addressing various aspects of care, such as education, manual lymphatic drainage, compression therapy, exercise programs, skincare, and lifestyle modifications, physiotherapists can significantly improve the quality of life for individuals living with elephantiasis-related lymphedema. Physiotherapy interventions aim to reduce swelling, enhance lymphatic circulation, prevent complications, and promote functional independence. However, it is important to emphasize that this condition is typically chronic and requires a long-term commitment from the patient and the healthcare team. Moreover, collaboration among healthcare professionals is essential to provide a comprehensive approach to care. Ultimately, physiotherapy is pivotal in empowering patients to manage their condition, minimize symptoms, and enhance their overall well-being while living with chronic lymphedema secondary to elephantiasis.
